# Comprehensive gene expression analysis of canine invasive urothelial bladder carcinoma by RNA-Seq

**DOI:** 10.1186/s12885-018-4409-3

**Published:** 2018-04-27

**Authors:** Shingo Maeda, Hirotaka Tomiyasu, Masaya Tsuboi, Akiko Inoue, Genki Ishihara, Takao Uchikai, James K. Chambers, Kazuyuki Uchida, Tomohiro Yonezawa, Naoaki Matsuki

**Affiliations:** 1 Department of Veterinary Clinical Pathobiology, Graduate School of Agricultural and Life Sciences, The University of Tokyo, Tokyo, Japan; 2 Veterinary Medical Center, Graduate School of Agricultural and Life Sciences, The University of Tokyo, Tokyo, Japan; 3 Department of Veterinary Pathology, Graduate School of Agricultural and Life Sciences, The University of Tokyo, Tokyo, Japan; 4 Anicom Specialty Medical Institute Inc., Tokyo, Japan

**Keywords:** Dog, Transitional cell carcinoma, Animal model, RNA sequencing

## Abstract

**Background:**

Invasive urothelial carcinoma (iUC) is a major cause of death in humans, and approximately 165,000 individuals succumb to this cancer annually worldwide. Comparative oncology using relevant animal models is necessary to improve our understanding of progression, diagnosis, and treatment of iUC. Companion canines are a preferred animal model of iUC due to spontaneous tumor development and similarity to human disease in terms of histopathology, metastatic behavior, and treatment response. However, the comprehensive molecular characterization of canine iUC is not well documented. In this study, we performed transcriptome analysis of tissue samples from canine iUC and normal bladders using an RNA sequencing (RNA-Seq) approach to identify key molecular pathways in canine iUC.

**Methods:**

Total RNA was extracted from bladder tissues of 11 dogs with iUC and five healthy dogs, and RNA-Seq was conducted. Ingenuity Pathway Analysis (IPA) was used to assign differentially expressed genes to known upstream regulators and functional networks.

**Results:**

Differential gene expression analysis of the RNA-Seq data revealed 2531 differentially expressed genes, comprising 1007 upregulated and 1524 downregulated genes, in canine iUC. IPA revealed that the most activated upstream regulator was PTGER2 (encoding the prostaglandin E_2_ receptor EP2), which is consistent with the therapeutic efficiency of cyclooxygenase inhibitors in canine iUC. Similar to human iUC, canine iUC exhibited upregulated ERBB2 and downregulated TP53 pathways. Biological functions associated with cancer, cell proliferation, and leukocyte migration were predicted to be activated, while muscle functions were predicted to be inhibited, indicating muscle-invasive tumor property.

**Conclusions:**

Our data confirmed similarities in gene expression patterns between canine and human iUC and identified potential therapeutic targets (PTGER2, ERBB2, CCND1, Vegf, and EGFR), suggesting the value of naturally occurring canine iUC as a relevant animal model for human iUC.

**Electronic supplementary material:**

The online version of this article (10.1186/s12885-018-4409-3) contains supplementary material, which is available to authorized users.

## Background

Urothelial carcinoma of the bladder is a common malignancy with 165,000 estimated global human deaths per year [1]. This cancer is heterogeneous and histologically classified into low-grade, superficial type and high-grade, invasive type. Low-grade tumors are more common (approximately 70%) and are usually associated with a favorable prognosis. In contrast, high-grade, muscle-invasive urothelial carcinoma (iUC) is less prevalent (approximately 30%) but has a high risk of death from distant metastasis [2]. Cisplatin-based chemotherapy is the standard first-line treatment of metastatic iUC and provides overall survival benefits [3]. Meanwhile, up to two-thirds of patients cannot tolerate the regimens due to nephrotoxicity [4]. There have been little advances in the treatment of iUC in the past 20 years. Thus, better management of iUC is urgently required.

Our understanding of various cancers has benefited greatly from comparative oncology using relevant animal models, especially mouse models. Several experimentally induced mouse models of bladder cancer have been established, including carcinogen-based models, engraftment models, and genetically engineered models. However, in contrast to many other cancer types, relatively few mouse models of bladder cancer have been described, and in particular, few models display muscle-invasive or metastatic cancer phenotypes [5]. Therefore, more suitable iUC animal models are crucial.

Naturally occurring iUC in pet dogs closely resembles human iUC with regard to clinical signs, histopathology, heterogeneity, metastatic behavior, disease progression, and response to cisplatin-based chemotherapy [6, 7]. Bladder carcinoma makes up approximately 2% of all spontaneous cancers in dogs, which is similar to its incidence in humans [1, 7]. However, unlike humans, the vast majority of canine bladder carcinomas are high-grade iUC (> 90% of cases); low-grade, superficial tumors are uncommon in dogs. Metastases of lymph nodes or distant regions (e.g., lungs, liver, kidneys) have been reported in approximately 15% of dogs at diagnosis and in 40–50% at death [6]. Moreover, clinical trials of canine iUC can be conducted in a relatively short period because dogs have a shorter life span than humans. Thus, dogs with iUC offer an ideal model to evaluate novel anti-cancer drugs. However, canine iUC has not been well characterized at the molecular level. Only one study, based on microarray analysis, has shown similarities in gene expression patterns between dogs and humans with iUC [8]. Although this previous study revealed the involvement of certain genes (TP53 and EGFR), other pathways related to carcinogenesis were not mentioned. To our knowledge, no detailed gene expression analyses using RNA sequencing (RNA-Seq) in canine iUC have been reported to date. RNA-Seq allows more sensitive detection of transcripts than microarrays in both dogs and humans [9, 10], contributing to a better understanding of molecular pathways in iUC.

In the present study, we performed a comprehensive gene expression analysis of canine iUC by RNA-Seq to identify key molecular pathways in canine iUC. The findings from dogs with iUC have the potential to improve our understanding of progression, diagnosis, and treatment of iUC in humans.

## Methods

### Ethics statement

The study protocol of tumor tissue sampling was approved by the animal care committee of the Veterinary Medical Center of the University of Tokyo, and written informed consent was obtained from all dog owners. The use of experimental animals in this study was approved by the animal care committee of the University of Tokyo (approval no. P11–530).

### Tissue samples

Tumor tissue specimens were obtained from 11 dogs with naturally occurring iUC by total cystectomy at the Veterinary Medical Center of the University of Tokyo. No cases received chemotherapy or radiation prior to tissue sampling. Each tumor tissue was macroscopically obtained by a pathologist with Japanese College of Veterinary Pathologists board certification (M.T.) and was fixed in 10% neutral buffered formalin for 24 h prior to embedding in paraffin. Histopathological examination confirmed a diagnosis of high-grade iUC for all cases. Each case was categorized by histology according to the World Health Organization criteria [11, 12]. Small pieces of tumor specimens were collected and snap-frozen in liquid nitrogen and stored at − 80 °C for RNA extraction. Urine samples were collected from all dogs using a urethral catheter, and BRAF V595E mutation was assessed by digital PCR assay [13].

Archived snap-frozen normal bladder tissues obtained from five healthy beagles euthanized for other experimental purposes were used as a control. This group included two females (all intact) and three males (all intact), aged 97–130 months (median, 112 months). These dogs exhibited no clinical signs and were not treated with any drugs. Routine urinalysis and blood examinations, including a complete blood count and measurements of blood urea nitrogen, creatinine, alanine aminotransferase, and alkaline phosphatase levels, showed no abnormalities.

### RNA-Seq

Total RNA was extracted from tumor and normal bladder tissues using the RNeasy Mini Kit (Qiagen, Valencia, CA). RNA integrity was examined with an Agilent 2100 Bioanalyzer (Agilent Technologies, Santa Clara, CA) and RNA integrity number (RIN) values of all samples were > 7. Sequencing libraries were prepared with the TruSeq Stranded mRNA Library Prep Kit for NeoPrep (Illumina, San Diego, CA). RNA-Seq (75-bp paired-end) using NextSeq 500 (Illumina) with the High Output Kit (Illumina) was conducted, and a minimum of 35 million read-pairs was generated for each sample.

Initial quality control of RNA-Seq data (FASTQ) for each sample was performed using the FastQC software (version 0.11.5; http://www.bioinformatics.babraham.ac.uk/projects/fastqc). FASTQ data were trimmed with Trimmomatic (version 0.36) [14]. Bowtie2 (version 2.2.9) [15] and TopHat (version 2.0.14) were used to map paired-end reads to canine genomes (CanFam3.1) and analyze mapping results to identify splice junctions between exons. Transcript abundance was estimated using Cufflinks [16] with gene transfer file (CanFam3.1.86). Fragments per kilobase of transcript per million mapped reads (FPKM) was imported into R (version 3.3.2), and principal component analysis (PCA) was conducted with genes for which the sum of FPKMs of all samples was > 10. Next, gene counts for each sample were imported into R for differential gene expression analysis with EdgeR [17, 18]. TMM normalization [19] and Tagwise dispersion (individual dispersion for each gene) were used to adjust for abundance differences across samples, and differentially expressed genes (DEGs) were extracted. The normalized gene counts were imported into Cluster3.0 and Java TreeView (version 1.1.6r4) for hierarchical clustering analysis and visualization. Enriched pathway and functional classification analyses of differences in expression of DEGs were performed using Ingenuity® Pathway Analysis (IPA®, Qiagen). The datasets used and/or analysed during the current study are available from the corresponding author on reasonable request and will also be available at the DDBJ Sequenced Read Archive repository (http://trace.ddbj.nig.ac.jp/dra/index_e.html) with accession number DRA005844.

### Quantitative real-time PCR

The mRNA expression levels of 5 DEGs extracted in this study were quantified by 2-step real-time RT-PCR (Thermal Cycler Dice Real Time System; Takara Bio, Shiga, Japan), as described previously [20, 21]. TATA-box binding protein (TBP) was used as a reference gene; primer pair sequences are shown in Additional file 1: Table S1.

### Immunohistochemistry

Immunohistochemistry (IHC) for EP2 was conducted on 4-μm-thick paraffin-embedded sections of the 15 iUC tissues including the 11 cases used for RNA-Seq analysis. A rabbit polyclonal antibody against EP2 (1:100 in dilution, Item No. 101750, Cayman Chemical, Ann Arbor, MI) was used and IHC was performed following previous studies [22, 23].

### Statistics

Statistical analyses were performed using JMP 9 (SAS Institute, Cary, NC). Fisher’s exact test was used to compare the sex distribution among dogs with iUC and healthy controls. The Mann–Whitney U test was used to compare age and mRNA expression of DEGs between dogs with iUC and healthy controls. Statistical significance was defined as *P* < 0.05.

## Results

### Clinical and pathological findings in dogs with iUC

All 11 cases had a histopathological diagnosis of high-grade iUC, and characteristics of each case are shown in Table 1. The dogs comprised 10 females (five intact and five neutered) and one intact male, aged 85–161 months (median, 145 months), with several breeds represented. There were no significant differences in sex and age between the iUC cases and healthy controls. According to the WHO TNM classification for canine urinary bladder cancer [24, 25], tumors in 8/11 (72.7%) dogs were classified as T2 tumors (muscle invasive) and those in 3/11 (27.3%) as T3 tumors (tumor invading neighboring organs). Iliac lymph node involvement was detected in 2/11 (18.2%) dogs. No distant metastasis (to the lung) was observed in any of the cases at diagnosis, while papillary tumors were observed in 8/11 (72.7%) dogs. Tumor grade was determined according to most anaplastic nuclear appearance [12]: tumors in 2/11 (18.2%) dogs were classified as grade 1 (well-differentiated cells), 6/11 (54.5%) as grade 2 (moderately differentiated cells), and 3/11 (27.3%) as grade 3 (anaplastic cells). Various types and degrees of inflammation were observed in each tumor microenvironment (Table 1). BRAF V595E mutation was detected in 6/11 (54.5%) cases.

**Table 1:** Clinical and histological characteristics of dogs with iUC and healthy control dogs

Case No.	Sex	Age (months)	Breed	TNM stage at diagnosis	Papillary histology	Grade (nuclear atypia)	Type (degree) of inflammation	BRAF mutation
iUC1	F	146	Wire Fox Terrier	T2N0M0	No	Grade 2	Lyn, Neu (mild)	–
iUC2	FS	122	Papillon	T2N0M0	Yes	Grade 2	Lym (moderate)	+
iUC3	F	85	Miniature Dachshund	T3N0M0	Yes	Grade 2	Lym (moderate)	+
iUC4	F	147	Papillon	T2N0M0	Yes	Grade 3	Lym (mild)	–
iUC5	FS	131	Miniature Dachshund	T3N0M0	Yes	Grade 3	Lym, Eo (moderate)	+
iUC6	F	122	Border Collie	T2N0M0	No	Grade 2	Lym (mild)	–
iUC7	FS	149	Miniature Dachshund	T2N1M0	No	Grade 3	Lyn, Neu (mild)	+
iUC8	F	105	French Bulldog	T2N0M0	Yes	Grade 3	Lym (moderate)	+
iUC9	M	161	Pembroke Welsh Corgi	T3N1M0	Yes	Grade 2	Lym (mild)	–
iUC10	FS	158	Toy Poodle	T2N0M0	Yes	Grade 2	Lym (severe)	+
iUC11	FS	145	Mongrel	T2N0M0	Yes	Grade 1	Lym (mild)	–
HC1	F	130	Beagle	–	–	–	–	–
HC2	F	112	Beagle	–	–	–	–	–
HC3	M	97	Beagle	–	–	–	–	–
HC4	M	122	Beagle	–	–	–	–	–
HC5	M	108	Beagle	–	–	–	–	–

*iUC* invasive urothelial carcinoma, *HC* healthy control, *F* female, *FS* female spayed, *M* male, *Lym* lymphocyte, *Neu* neutrophil, *Eo* eosinophil

### Principal component analysis

**Fig. 1:**
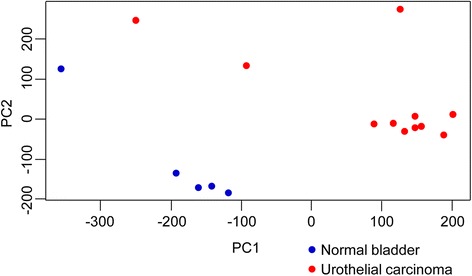
Principal component analysis (PCA) plot of canine iUC and normal bladder. The PCA plot shows clear separation between canine iUC (red) and normal bladder (blue) samples

The differences in gene expression profiles between normal bladder and canine iUC samples are illustrated in the PCA plot (Fig. 1), which clearly separates normal bladder from tumor samples. A previous study revealed that the BRAF V595E mutation is observed at significantly high rates (approximately 80%) in canine iUC [13]. However, the pathological significance of this mutation remains unclear. Thus, we used PCA to assess the clustering of canine iUC cases with or without BRAF V595E mutation. The PCA plot failed to show a clear separation of cases with or without BRAF mutation (Additional file 2: Figure S1).

### Differential expression analysis

**Fig. 2:**
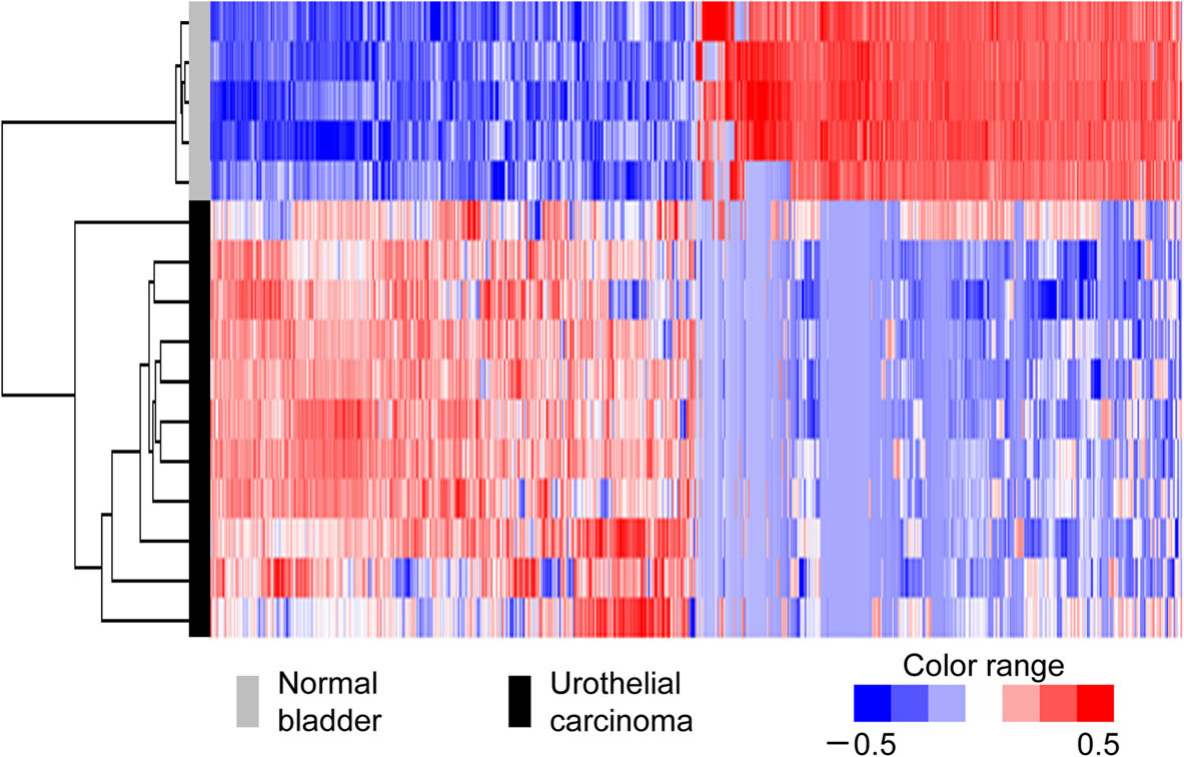
Hierarchical clustering of differentially expressed genes between canine iUC and normal bladder. Genes indicated in blue are down-regulated, while genes indicated in red are upregulated

Differential expression analysis revealed several genes that were differentially regulated in iUC cases when compared with normal controls with a q-value < 0.01. In total, 2531 DEGs showed significant changes between normal bladder and iUC samples. Of these, 1007 DEGs were upregulated and 1524 were downregulated in iUC dogs with a fold-change ≥2. The 500 most strongly up- and downregulated DEGs are shown in Additional file 3: Table S2 and Additional file 4: Table S3, respectively. Hierarchical clustering of these DEGs showed a similar gene expression pattern among the 11 canine iUC tissues and a clear separation of normal and tumor tissues (Fig. 2). When hierarchical clustering was conducted using the genes that were shown to divide the human iUC patients into subgroups [26], no clear separation was observed between normal controls and canine iUCs (data not shown). Additional hierarchical clustering was conducted using 388 genes that were shown to divide the dogs with iUC into subgroups [8], and three clusters were identified: those composed of five normal controls, two iUCs (TCC1 and TCC11), or remaining nine iUCs (Additional file 5: Figure S2).

To validate our findings from the RNA-Seq analysis, we examined mRNA expression levels of Top 5 DEGs (CHST4, PIGR, S100A14, ADGRF1, and AGR2) by quantitative PCR and confirmed the trend observed in the RNA-Seq data, whereby there was a significant increase in dogs with iUC compared to that in healthy controls (Additional file 6: Figure S3).

### Pathway analysis

Enriched pathway and functional classification analyses of DEGs were performed using IPA. For simplicity, IPA analysis was conducted with the 500 most strongly up- and downregulated DEGs. Extracted upstream regulators associated with these DEGs are shown in Table 2 and Additional file 7: Table S4. Relative to those in normal bladder, the most significant upstream regulators predicted to be activated in canine iUC were PTGER2 and ERBB2, whereas those predicted to be inhibited were Irgm1, TP53, let-7, and ZFP36 (Table 2). The transcription factors CCND1, FOXM1, E2F3, FOXO1, and JUN as well as proinflammatory and angiogenesis cytokines IL6, IL1B, CSF2, TNF, IFNG, IL22, HGF, and Vegf were among the top 30 upstream regulators predicted to be activated.

**Table 2:** Top 30 upstream regulators of differentially expressed genes in canine iUC

Upstream regulator	Molecule type	Predicted activation state	Bias-corrected z-score	*P*-value of overlap
PTGER2	G-protein coupled receptor	Activated	4.735	1.15E-17
ERBB2	Kinase	Activated	4.679	9.58E-17
Irgm1	Other	Inhibited	−4.175	9.40E-16
IL6	Cytokine	Activated	3.46	3.32E-15
IL1B	Cytokine	Activated	4.071	3.01E-13
CSF2	Cytokine	Activated	5.706	8.53E-13
CCND1	Transcription regulator	Activated	4.073	2.25E-12
TP53	Transcription regulator	Inhibited	−2.977	1.41E-11
TNF	Cytokine	Activated	4.168	1.57E-11
FOXM1	Transcription regulator	Activated	4.079	3.90E-09
HGF	Growth factor	Activated	3.956	6.87E-09
E2F3	Transcription regulator	Activated	3.186	7.04E-09
FOXO1	Transcription regulator	Activated	3.643	1.21E-08
NFkB (complex)	Complex	Activated	3.65	2.39E-08
IFNG	Cytokine	Activated	3.536	2.72E-08
let-7	MicroRNA	Inhibited	−4.119	3.16E-08
RABL6	Other	Activated	3.197	3.28E-08
JUN	Transcription regulator	Activated	4.005	4.11E-08
IL1A	Cytokine	Activated	3.265	1.94E-07
E2f	Group	Activated	3.226	2.36E-07
Ap1	Complex	Activated	2.789	2.38E-07
IL22	Cytokine	Activated	3.199	4.46E-07
Vegf	Group	Activated	3.595	4.97E-07
MAP2K3	Kinase	Activated	2.776	5.32E-07
NfkB-RelA	Complex	Activated	2.107	5.68E-07
ERK1/2	Group	Activated	4.027	1.03E-06
EGFR	Kinase	Activated	3.463	1.17E-06
IFI16	Transcription regulator	Activated	2.427	1.44E-06
E2F2	Transcription regulator	Activated	2.634	1.82E-06
ZFP36	Transcription regulator	Inhibited	−3.606	2.12E-06

Predicted activity based on gene expression values in canine iUC relative to normal bladder. A z-score > 2 indicates activation, while a z-score < −2 indicates inhibition

We further performed IPA to examine which biological processes are associated with canine iUC. IPA revealed that gene expression in canine iUC tissues was associated with biological processes involving cancer, neoplasia of epithelial tissue, tumorigenesis of tissue, proliferation of cells and cell movement of leukocytes (Table 3). Mammary tumor gene expression was also predicted to be activated, while gene expression related to muscle function and contractility was predicted to be inhibited.

**Table 3:** Top biological functions of differentially expressed genes in canine iUC

Biological functions	Predicted activation state	Bias-corrected z-score	*P*-value
Cancer	Activated	2.051	2.08E-22
Epithelial cancer	Activated	2.834	5.93E-21
Neoplasia of epithelial tissue	Activated	2.221	7.60E-21
Tumorigenesis of tissue	Activated	2.21	1.10E-20
Abdominal neoplasm	Activated	2.057	2.58E-19
Proliferation of cells	Activated	4.057	3.16E-14
Cell movement of leukocytes	Activated	2.678	6.35E-10
Function of muscle	Inhibited	−2.234	9.06E-09
Mammary tumor	Activated	2.228	3.46E-08
Contractility of muscle	Inhibited	−3.244	4.22E-08

Predicted activity based on gene expression values in canine iUC relative to healthy bladder. A z-score > 2 indicates activation, while a z-score < −2 indicates inhibition

### Immunohistochemistry for EP2

**Fig. 3:**
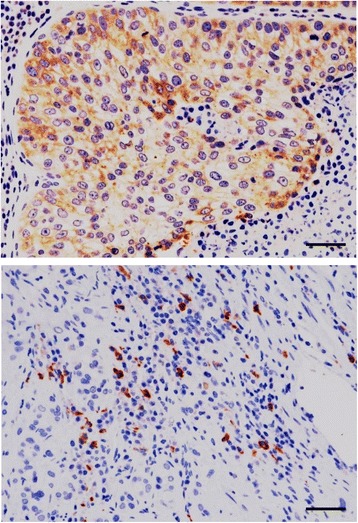
Detection of EP2 in canine iUC tissues by immunohistochemistry. EP2 expression was observed in the membrane and cytoplasm of primary tumor cells (upper) and peritumoral inflammatory cells (lower). Bar = 50 μm

Our RNA-Seq analysis revealed that PTGER2 was the most activated upstream regulator in canine iUC (*P* < 1.15E-17, Table 2). To further examine protein expression, IHC for EP2, which is encoded by PTGER2, was performed on tumor sections from 15 dogs with histopathologically confirmed iUC. EP2 expression was observed in primary tumor cells and peritumoral inflammatory cells (Fig. 3); EP2 expression was detected in tumor and inflammatory cells in 11/15 (73.3%) and 2/15 (13.3%) of dogs, respectively. In contrast, normal epithelial cells did not express EP2 (data not shown).

## Discussion

In this study, we applied a transcriptome sequencing approach to identify gene expression characteristics of canine iUC. We detected 2531 DEGs, and hierarchical clustering of these DEGs showed a clear separation of normal and tumor tissues. Using genes that were previously shown to divide the canine iUC into subgroups [8], the present study also identified two distinct subgroups within dogs with iUC. Furthermore, pathway analysis revealed a number of upstream regulators of the DEGs in canine iUC, providing insights into disease biology and potential targets for therapeutic intervention.

PTGER2, encoding prostaglandin E_2_ (PGE_2_) receptor 2 (also known as EP2), was the most activated upstream regulator in canine iUC. In addition, we confirmed that EP2 protein was strongly expressed in tumor cells and surrounding inflammatory cells but not in normal epithelial cells. PGE_2_ is a major arachidonic acid-derived metabolite that is abundantly produced by cyclooxygenase-2 (COX-2) upon inflammation [27]. The biological effects of PGE_2_ are mediated through four types of the receptor, EP1–4 [28]. Upregulation of COX-2 accompanied by high levels of PGE_2_ is common in a variety of tumor tissues [29]. Previous studies have shown that elevated expression of COX-2 and increased levels of PGE_2_ are observed in both human and canine bladder cancer [30–35]. PGE_2_–EP2 signaling promotes tumor development via multiple mechanisms, including stimulatory effects on cell proliferation, survival, invasion, angiogenesis, and metastasis [36]. A recent study demonstrated that PGE_2_ induces programmed cell death protein ligand 1 expression in tumor-associated macrophages and myeloid-derived suppressor cells, leading to the inhibition of anti-tumor immunity [37]. Furthermore, PGE_2_ is involved in resistance to chemotherapy by promoting repopulation of cancer stem cells in bladder cancer [38]. In addition, COX-2 inhibitors consistently exert anti-tumor effects on bladder cancer in both dogs and humans [7, 24, 39]. These findings suggest that the COX-2/PGE_2_/EP2 pathway plays a role in tumorigenesis and progression of bladder cancer.

ERBB2 (also known as HER2) was the second most activated upstream regulator in canine iUC. HER2 is a membrane-bound receptor tyrosine kinase belonging to the epidermal growth factor receptor (EGFR) family and is implicated as an oncogene in many human cancers [40]. Notably, HER2 is amplified and overexpressed in one-third of patients with breast cancer and is associated with poor prognosis [41]. HER2 overexpression facilitates excessive dimer formation, driving oncogenic cell survival, proliferation, apoptosis suppression, and angiogenesis [40, 42]. Thus, a number of clinical trials of HER2-targeted therapies in patients with breast cancer have been conducted, showing increased survival in both the metastatic and early-stage settings of the disease [40, 43–45]. Recently, The Cancer Genome Atlas project reported mutation or amplification of ERBB2 in a subset of human iUC [26]. Another study also identified activating ERBB2 mutations in the absence of gene amplification in human iUC [46]. These observations suggest that HER2-targeted therapy might also be effective for the treatment of iUC. Clinical trials to evaluate the efficacy and safety of HER2-targeted drugs in patients with bladder cancer are ongoing [47]. Our data indicate that HER2 is involved in disease progression and is a therapeutic target in canine iUC, as is the case with humans.

Pathways of several transcription factors (CCND1, FOXM1, E2F3, FOXO1, and JUN) were enriched in canine iUC. Of these, CCND1, which encodes cyclin D1, plays a critical role in the cell cycle machinery (e.g., G1-S transition) and is considered a promising target for cancer therapy [48]. Proinflammatory and angiogenesis cytokines (IL6, IL1, CSF2, TNF, IFNG, IL22, HGF, and Vegf) were also enriched in canine iUC. Our data support that aberrant inflammation and neovascularization occur in the tumor environment, and that anti-inflammatory and anti-angiogenic drugs targeting these cytokines have therapeutic potential for canine iUC [49]. Recently, immune-checkpoint inhibitors targeting the programmed death 1/programmed death-ligand 1 (PD-1/PD-L1), atezolizumab and nivolumab, have been approved by the Food and Drug Administration (FDA) in patients with advanced bladder cancer [50]. Although our study did not detect PD-L1 (CD274) within the 2513 DEGs, immune-checkpoint inhibitors might be effective against canine iUC.

By contrast, this study predicted that the pathways of Irgm1, TP53, let-7, and ZFP36 are inhibited in canine iUC. Irgm1 is a member of the interferon-γ (IFN-γ)-inducible GTPase family and plays a role in innate immunity by regulating autophagy [51]. Recent studies have reported that Irgm1 enhances tumorigenesis and metastasis of melanoma in mice [52, 53]. High concentrations of IFN-γ are toxic to neighbor cells; however, Irgm1 protects immune cells that produce IFN-γ from autophagic cell death, thereby promoting the expansion of CD4^+^ T cells [54]. Although the significance of Irgm1 in canine iUC is unclear, inhibition of the pathway might prevent anti-tumor immunity from eliminating cancer cells through enhancement of IFN-γ-induced immune cell death.

TP53 is one of the most important tumor-suppressor genes and plays a role in cell cycle arrest and apoptosis in response to DNA damage [55]. In humans, about half of all tumor types including bladder cancer possess alterations in TP53 or its upstream/downstream genes [26, 56]. In dogs, TP53 mutations are also observed in many types of tumors, such as lymphoma [57], osteosarcoma [58, 59], and mammary tumor [60, 61]. In canine iUC, the presence of TP53 mutation is deduced by immunohistochemistry (IHC). The mutated p53 protein, with a prolonged half-life, is considered to result in positive immunoreactivity of neoplastic cell nuclei, and thus can be detected by IHC [62]. However, TP53 mutations have not been directly confirmed in canine iUC by DNA sequencing analysis. Considering that the TP53 pathway was inhibited in this study, genetic and/or epigenetic abnormalities of TP53 may be present in canine iUC.

let-7 is a founding member of the microRNA (miRNA) family, postulated to function as a tumor suppressor gene by negatively regulating the post-transcriptional expression of multiple oncogenes including Ras, Myc, and Hmga2, as well as other cell cycle regulator genes in humans [63–65]. Reduced expression of let-7 is often observed and associated with poor prognosis in many human cancers, particularly in lung carcinoma [66]. Furthermore, let-7 has an attractive potential for the treatment of cancers; for example, intranasal let-7 administration reduced tumor formation in a mouse model of lung cancer [67]. As the let-7 pathway was significantly inhibited while the Ras pathway was significantly activated in canine iUC (Additional file 7: Table S4), exogenous let-7 administration may restore the reduced expression of let-7 and may be a potent therapeutic strategy.

ZFP36 encodes a tandem Cys-Cys-Cys-His zinc finger protein (also known as tristetraprolin) that binds to and promotes degradation of AU-rich elements in the 3′-untranslated regions of certain mRNAs [68]. COX-2 is a well-established target of ZFP36 [69, 70], and in this study, we found that dogs with iUC exhibited suppression of the ZFP36 pathway. Thus, overexpression of COX-2 in canine iUC [30, 31] could be attributed to the inhibition of the ZFP36 pathway and subsequent lack of rapid mRNA degradation.

Highly expressed genes present in canine iUC were associated with cancer and leukocyte migration, whereas downregulated genes were associated with muscle function. Decreased expression of muscle-related genes may result in a muscle-invasive phenotype. In addition, gene sets enriched in canine iUC were associated with mammary tumors, consistent with previous studies whereby signatures of human bladder cancer are similar to that of breast cancer [26, 71].

Several biomarkers can be used as a screening test for bladder cancer in dogs as well as humans [72]. Urinary bladder tumor antigen (BTA) test is an agglutination reaction that detects complexes of basement membrane proteins degraded by proteinases of urothelial neoplasms [73]. The present study revealed that the mRNA expressions of matrix metalloproteinase 7 (MMP7), MMP9, MMP12, and MMP13 were significantly upregulated in canine iUC (Additional file 3: Table S2). A recent study observed significantly high rates of the BRAF V595E mutation (approximately 80%) in canine iUC and prostate cancer [13]. In our study, this mutation was detected in 6/11 (54.5%) cases, although no clear separation was observed between dogs with or without BRAF mutation in a PCA plot. A possible reason may be that the sample size was too small to detect a significant correlation; therefore, more extensive studies are needed to clarify the role of BRAF mutation in canine iUC.

One limitation of this study is that the healthy control dogs were not well matched to the iUC cases by sex and breed, although there was no significant difference in the sex distribution between the two groups. In the pathway analysis of this study, sex hormone-associated genes were not observed in the top 500 upstream regulators of DEGs (Additional file 7: Table S4). Therefore, it appears that gender bias did not impact the RNA-Seq data. Since it was impossible to obtain fresh bladder tissues from various breeds matched to the clinical cases, all healthy control dogs were the same breed, and this could lead to erroneous results related to the expression of some genes that differ between breeds. Further studies will be necessary to examine breed differences in bladder gene expression.

## Conclusions

In conclusion, alteration of multiple pathways in canine iUC was uncovered by RNA-Seq analysis. Upstream regulators of the altered pathways documented here are potential therapeutic and diagnostic targets for canine iUC. Our data confirmed the similarities in gene sets between dogs and humans, indicating the value of spontaneous canine iUC as a relevant animal model for human bladder cancer.

## Additional files

Additional file 1: **Table S1.** Sequences of the oligonucleotide primers used for quantitative PCR. (XLSX 11 kb)

Additional file 2: **Figure S1.** Principal component analysis (PCA) plot of canine iUC with or without BRAF V595E mutation. The PCA plot did not show clear separation between cases with (red) or without (blue) the BRAF mutation. (TIF 4649 kb)

Additional file 3: **Table S2.** Top 500 upregulated differentially expressed genes (DEGs) in canine iUC. (XLSX 26 kb)

Additional file 4: **Table S3.** Top 500 downregulated differentially expressed genes (DEGs) in canine iUC. (XLSX 25 kb)

Additional file 5: **Figure S2.** Hierarchical clustering of the genes that were previously shown to divide dogs with iUC into subgroups. Analysis yielded three clusters composed of five healthy controls, those of two dogs with iUC (iUC1 and iUC11), and those of the remaining nine iUC cases. Genes indicated in blue are down-regulated, while genes indicated in red are upregulated. (TIF 7001 kb)

Additional file 6: **Figure S3.** mRNA expression of top 5 DEGs in the bladder of healthy dogs and dogs with iUC. TBP was used as an internal control. Open and closed circles represent healthy dogs and dogs with iUC, respectively. *P < 0.05, **P < 0.01. (TIF 7016 kb)

Additional file 7: **Table S4.** Top 500 upstream regulators of differentially expressed genes in canine iUC. Predicted activity based on gene expression values in canine iUC relative to normal bladder. A z-score > 2 indicates activation, while a z-score < − 2 indicates inhibition. (XLSX 17 kb)

## Abbreviations

COX: Cyclooxygenase
DEGs: Differentially expressed genes
EGFR: Epidermal growth factor receptor
IFN-γ: Interferon-γ
iUC: Invasive urothelial carcinoma
miRNA: MicroRNA
PCA: Principal component analysis
PGE_2_: Prostaglandin E_2_
RNA-Seq: RNA sequencing
