# Supplementary figures and images for: Comprehensive gene expression analysis of canine invasive urothelial bladder carcinoma by RNA-Seq
Source: BMC Cancer. 2018 Apr 27;18:472. doi: 10.1186/s12885-018-4409-3 (PMC5921755; doi:10.1186/s12885-018-4409-3)

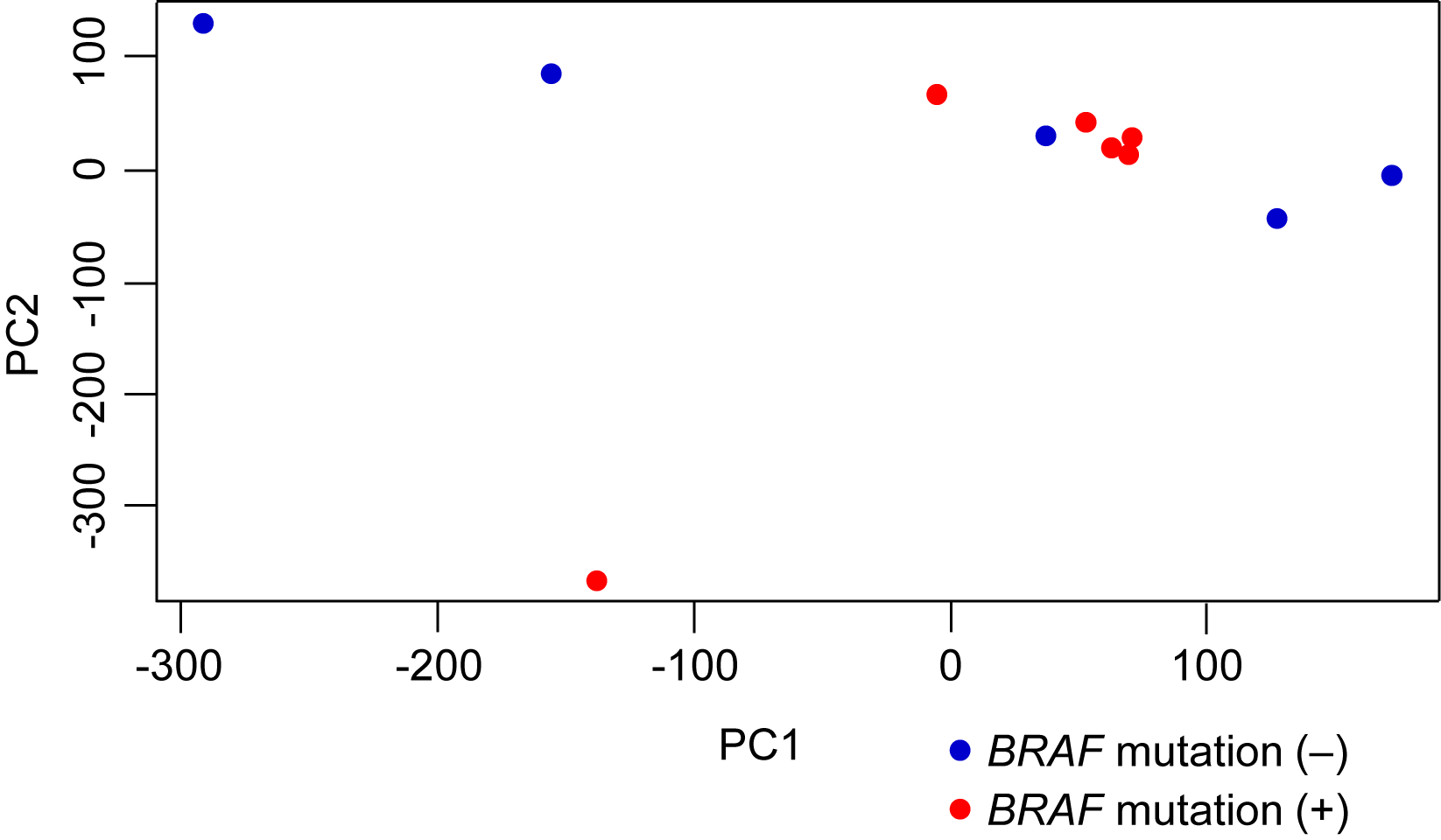

Supplement: Supplementary file 2 — Figure S1. Principal component analysis (PCA) plot of canine iUC with or without BRAF V595E mutation. The PCA plot did not show clear separation between cases with (red) or without (blue) the BRAF mutation. (TIF 4649 kb) [file 12885_2018_4409_MOESM2_ESM.tif]

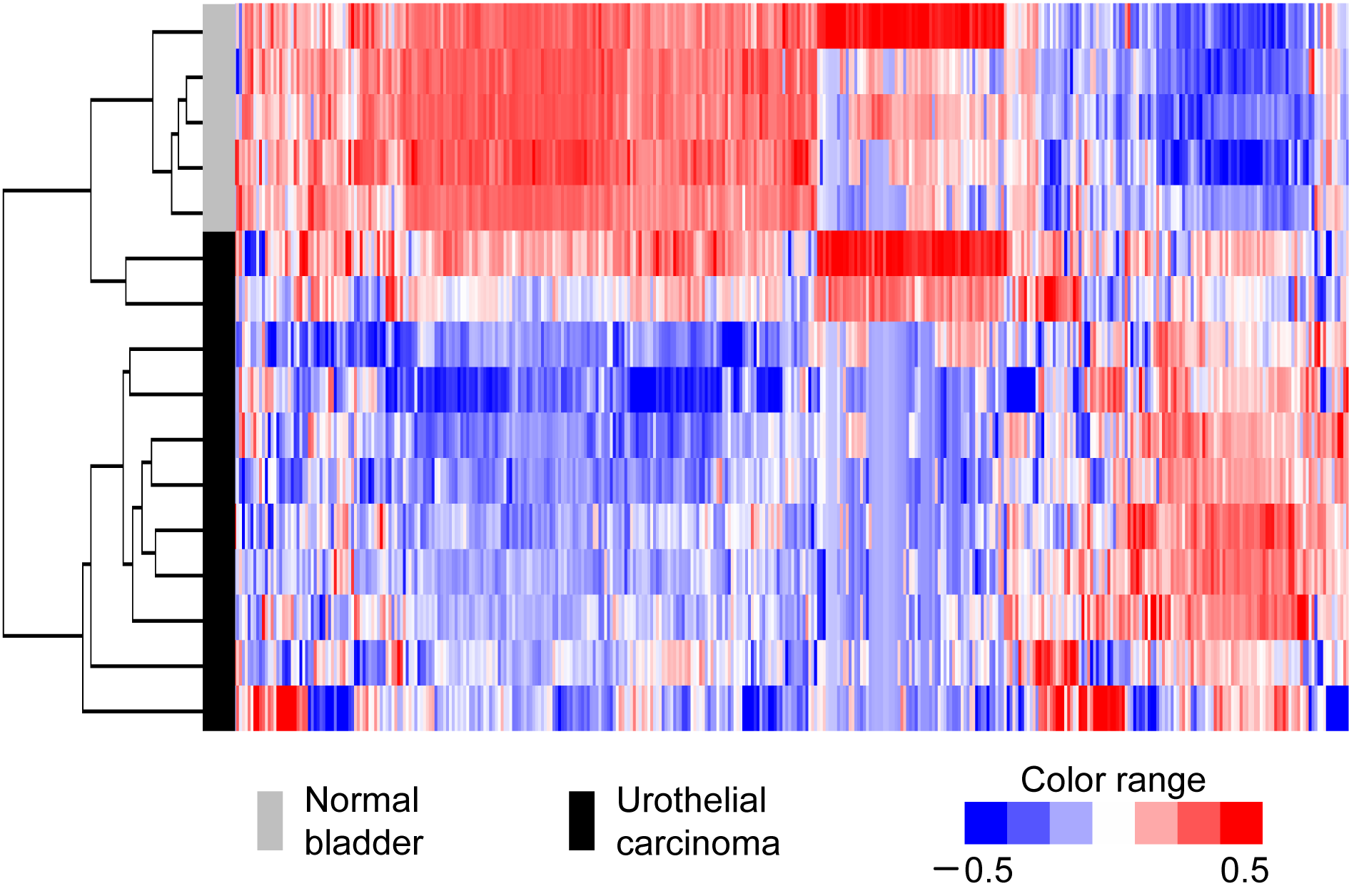

Supplement: Supplementary file 5 — Figure S2. Hierarchical clustering of the genes that were previously shown to divide dogs with iUC into subgroups. Analysis yielded three clusters composed of five healthy controls, those of two dogs with iUC (iUC1 and iUC11), and those of the remaining nine iUC cases. Genes indicated in blue are down-regulated, while genes indicated in red are upregulated. (TIF 7001 kb) [file 12885_2018_4409_MOESM5_ESM.tif]

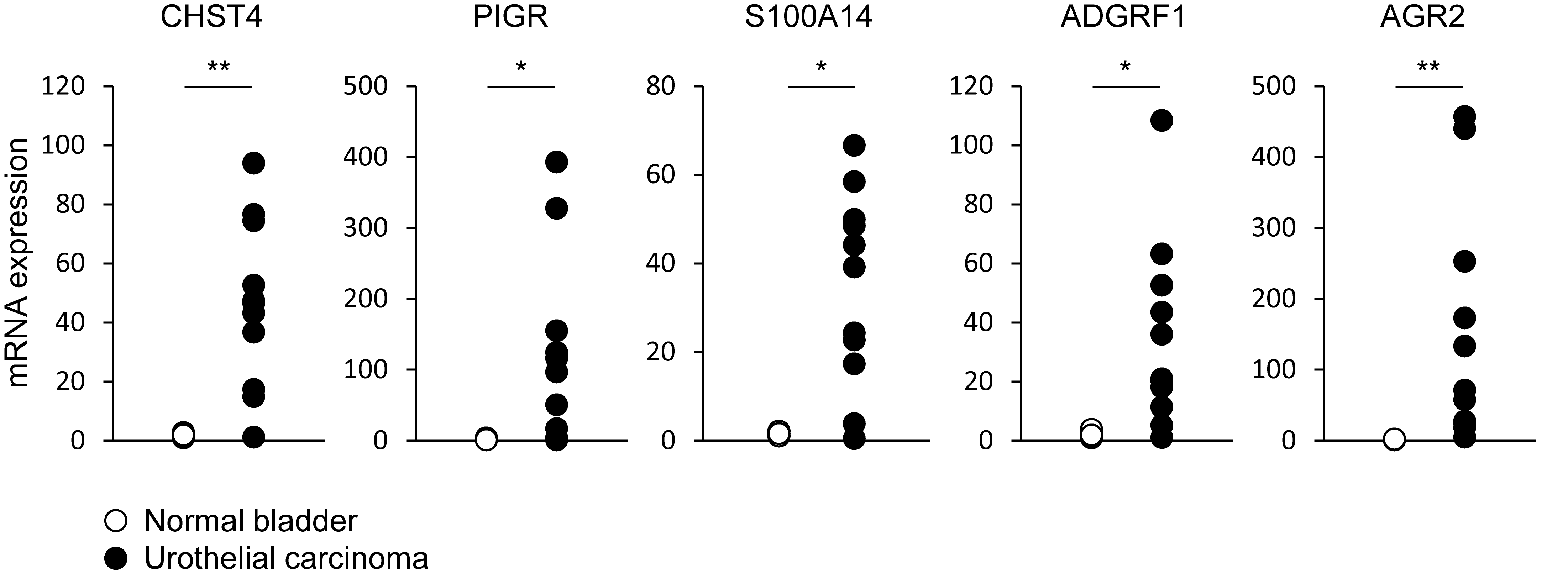

Supplement: Supplementary file 6 — Figure S3. mRNA expression of top 5 DEGs in the bladder of healthy dogs and dogs with iUC. TBP was used as an internal control. Open and closed circles represent healthy dogs and dogs with iUC, respectively. *P < 0.05, **P < 0.01. (TIF 7016 kb) [file 12885_2018_4409_MOESM6_ESM.tif]
